# Schnyder corneal dystrophy and associated phenotypes caused by novel and recurrent mutations in the *UBIAD1* gene

**DOI:** 10.1186/s12886-018-0918-8

**Published:** 2018-09-17

**Authors:** Cerys J. Evans, Lubica Dudakova, Pavlina Skalicka, Gabriela Mahelkova, Ales Horinek, Alison J. Hardcastle, Stephen J. Tuft, Petra Liskova

**Affiliations:** 1 UCL Institute of Ophthalmology, London, UK; 2 Research Unit for Rare Diseases, Department of Paediatrics and Adolescent Medicine, First Faculty of Medicine, Charles University and General University Hospital in Prague, Ke Karlovu 2, 128 08 Prague 2, Czech Republic; 3 Department of Ophthalmology, First Faculty of Medicine, Charles University and General University Hospital in Prague, Prague, Czech Republic; 4 Department of Ophthalmology, Second Faculty of Medicine, Charles University and Motol University Hospital, Prague, Czech Republic; 5 3rd Department of Medicine, Department of Endocrinology and Metabolism, First Faculty of Medicine, Charles University and General University Hospital in Prague, Prague, Czech Republic; 6 Institute of Biology and Human Genetics, First Faculty of Medicine, Charles University and General University Hospital in Prague, Prague, Czech Republic; 7 Moorfields Eye Hospital, London, UK

**Keywords:** Schnyder corneal dystrophy, *UBIAD1*, Novel mutation, De novo, Crystalline deposits, Confocal microscopy, Spectral domain optical coherence tomography

## Abstract

**Background:**

The purpose of this study was to identify the genetic cause and describe the clinical phenotype of Schnyder corneal dystrophy (SCD) in six unrelated probands.

**Methods:**

We identified two white Czech, two white British and two South Asian families with a clinical diagnosis of SCD. Ophthalmic assessment included spectral domain optical coherence tomography (SD-OCT) of one individual with advanced disease, and SD-OCT and confocal microscopy of a child with early stages of disease. *UBIAD1* coding exons were amplified and Sanger sequenced in each proband. A fasting serum lipid profile was measured in three probands. Paternity testing was performed in one family.

**Results:**

A novel heterozygous c.527G>A; p.(Gly176Glu) mutation in *UBIAD1* was identified in one Czech proband. In the second Czech proband, aged 6 years when first examined, a previously described de novo heterozygous c.289G>A; p.(Ala97Thr) mutation was found. Two probands of South Asian descent carried a known c.305G>A; p.(Asn102Ser) mutation in the heterozygous state. Previously reported heterozygous c.361C>T; p.(Leu121Phe) and c.308C>T; p.(Thr103Ile) mutations were found in two white British families. Although crystalline deposits were present in all probands the affected area was small in some individuals. Corneal arcus and stromal haze were the most prominent phenotypical feature in two probands. In the Czech probands, SD-OCT confirmed accumulation of reflective material in the anterior stroma. Crystalline deposits were visualized by confocal microscopy. Mild dyslipidemia was found in all three individuals tested.

**Conclusion:**

Although de novo occurrence of mutations in *UBIAD1* is extremely rare, SCD should be considered in the differential diagnosis of bilateral corneal haze and/or crystal deposition, especially in children.

## Background

Schnyder corneal dystrophy (SCD; MIM #121800) is a rare autosomal dominant disorder characterized by bilateral corneal opacification due to an accumulation of unesterified cholesterol and phospholipids in the corneal stroma [1]. Approximately 50% of individuals have crystalline deposits [2]. An association with genu valgum and systemic hyperlipidemia has also been reported [3].

SCD is caused by mutations in the *UBIAD1* gene (MIM *611632), encoding a membrane-embedded UbiA prenyltransferase domain-containing protein which catalyses the Mg^2+^-dependent transfer of a hydrophobic polyprenyl chain onto a variety of acceptor molecules, including vitamin K and coenzyme Q [1, 4, 5]. At least 26 mutations that cause SCD have been identified to date [6].

In this study we report the clinical and genetic investigation of six probands of white and South Asian origin.

## Methods

### Clinical examination

The study was approved by the relevant research ethics committees and adhered to the tenets of the Helsinki Declaration. Previously unreported probands from six families with a clinical diagnosis of SCD were investigated; two were recruited in the Czech Republic and four in the UK (Table 1). Family history of SCD was documented and available family members were invited to participate.

**Table 1:** Demographic and clinical data of six probands with Schnyder corneal dystrophy

No	Ethnicity	Family history	*UBIAD1* mutation	Age (when recruited)/gender	BCVA		Corneal phenotype	Chol (mmol/l)	HDL (mmol/l)	LDL (mmol/l)	TG (mmol/l)	Other relevant clinical data
					LE	RE						
1	White Czech	Y	c.527G>A p.(Gly176Glu)	36/M	0.6	0.7	Subepithelial central and mid-peripheral crystals in a ring pattern, minimal corneal arcus	**5.72**	1.58	**3.21**	1.51	
2	White Czech	N	c.289G>A p.(Ala97Thr)	6/F	0.6	0.5	Subepithelial mid-peripheral and mid-stromal crystals in a ring pattern	**4.89**	1.31	2.66	**2.05**	
3	White British	Not known	c.361C>T p.(Leu121Phe)	10/M	0.3	0.3	Mid-stromal central crystals	**5.00**	1.90	UA	1.80	Amblyopia in BE Lamellar keratoplasty in RE and LE at the age of 10 and 12 years
4	White British	Y	c.308C>T p.(Thr103Ile)	54/F	0.5	0.66	Central stromal haze, arcus, few mid-peripheral subepithelial crystals	UA*	UA	UA	UA	
5	South Asian	Y	c.305G>A p.(Asn102Ser)	37/F	0.66	0.66	Diffuse stromal haze, few subepithelial mid-peripheral crystals	UA	UA	UA	UA	Knee deformities, scoliosis, learning difficulties
6	South Asian	Y	c.305G>A p.(Asn102Ser)	40/F	0.5	0.66	Central stromal haze, arcus, few subepithelial mid-peripheral crystals	UA	UA	UA	UA	

*BCVA* best corrected visual acuity, *LE* left eye, *RE* right eye, *BE* both eyes, *M* male, *F* female, *Chol* total cholesterol, *HDL* high density lipoprotein, *LDL* low density lipoprotein, *TG* triglycerides, *- elevated but value not known, *UA* unavailable data

Elevated values are shown in bold

Ophthalmic examination included best corrected Snellen visual acuity (BCVA) converted to decimal values, intraocular pressure, and fundal examination after pupil dilation. We performed corneal imaging of probands 1 and 2 using spectral domain optical coherence tomography (SD-OCT) (Spectralis; Heidelberg Engineering GmbH). Proband 2 also underwent scanning slit confocal microscopy equipped with a non-applanating 40× immersion objective lens (Confoscan 3.0; Nidek Technologies, Viconza, Italy) [7].

We measured the fasting serum lipid profile of three probands and recorded the levels of total cholesterol, high and low-density lipoproteins, and triglycerides. The presence of joint deformity, scoliosis or learning difficulty was based on self-reported symptoms.

### Molecular genetic analysis

Genomic DNA from probands and any additional available family members was extracted from venous blood samples using a Gentra Puregene blood kit (Qiagen, Hilden, Germany) or from saliva using an Oragene kit (Oragene OG-300, DNA Genotek, Canada). We then performed PCR amplification and Sanger sequencing of the two *UBIAD1* coding exons and exon/intron boundaries (primer sequences and conditions are listed in Table 2). Variants were annotated against the reference sequence for transcript NM_013319.2. Mutation description followed standard nomenclature guidelines (http://varnomen.hgvs.org/) starting with nucleotide numbering c.1 at the A of the ATG translation initiation codon. Pathogenicity was evaluated in silico by six different algorithms (PROVEAN [8], SNPs&GO [9], MutPred [10], SIFT [11], PolyPhen-2 [12] and MutationTaster [13]). We also performed paternity testing in family 2, using a previously published set of markers [14]. The population frequency of variants was determined by the Genome Aggregation Database (gnomAD), which provides sequencing data from more than 123,136 exomes and 15,496 genomes from unrelated individuals of various ethnic backgrounds [15], and 2500 Czech control chromosomes available through the next generation sequencing projects of the Czech National Center for Medical Genomics (https://ncmg.cz/en).

**Table 2:** Primer sequences and condition used for PCR and Sanger sequencing of *UBIAD1* gene

Target	Forward primer	Reverse Primer	Size (bp)	Enzyme	Annealing Temp
exon 1	CCGTCCTTCCTCCTTCCC	AAGCCACCTTTGACATCCCT	700	GoTaqGreen	65 °C
exon 2	CCACCTGCACAGTCTAAGGA	CTGCCAAATCACATTCCTTCCT	689	GoTaqGreen	60 °C

## Results

**Fig. 1:**
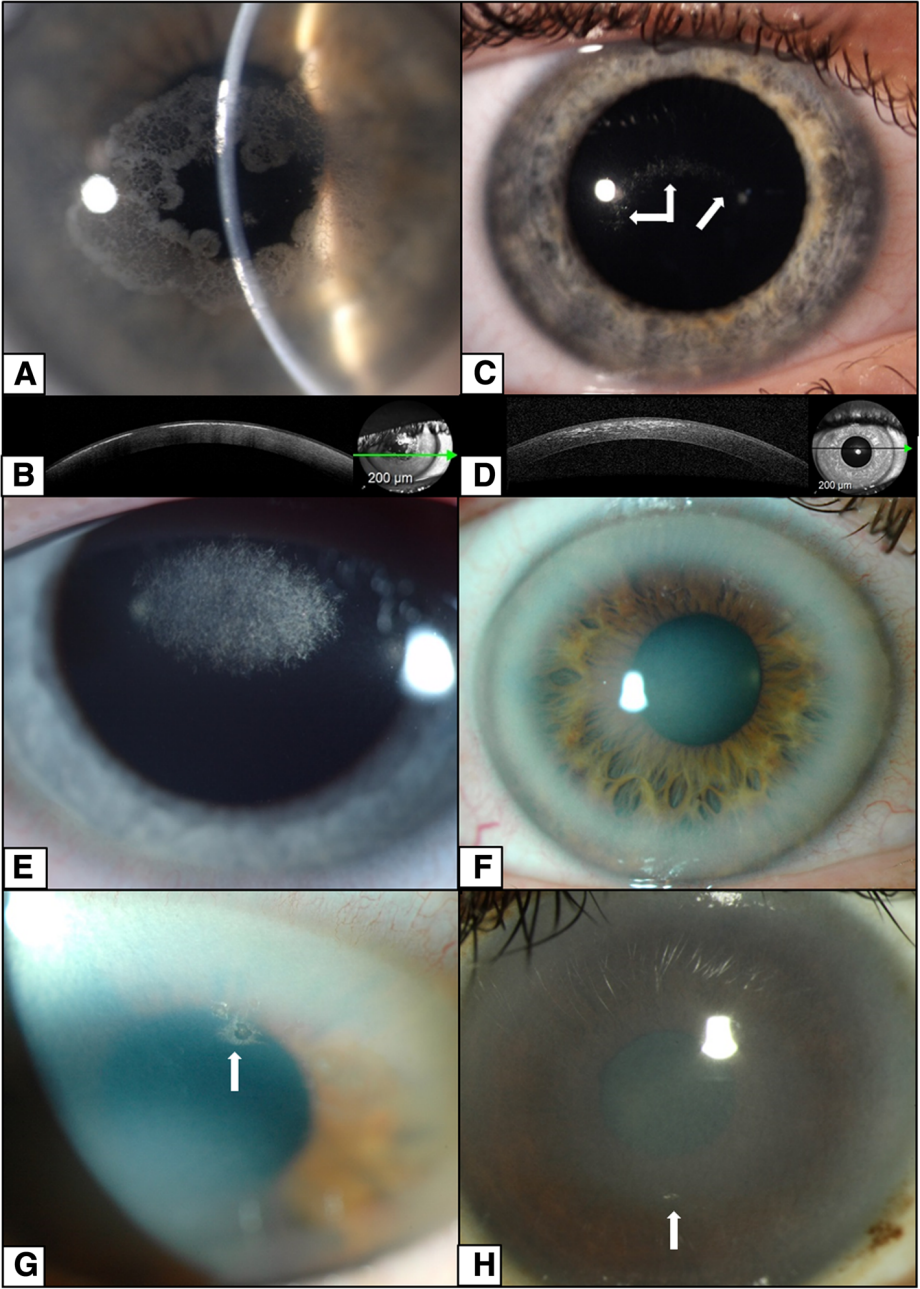
Corneal phenotype observed in five probands with Schnyder corneal dystrophy. Ring of prominent superficial crystalline deposits in proband 1 aged 36 years (**a**), also documented by SD-OCT as a discontinuous hyper-reflective line beneath the epithelium and within the anterior corneal stroma (**b**). Discrete crystalline deposits in proband 2 aged 8 years (**c**) and more scattered opacities on SD-OCT (**d**). Central mid-stromal crystalline deposits in proband 3 aged 10 years (**e**). Diffuse stromal haze with prominent arcus in proband 4 aged 54 years (**f**, **g**) and proband 5 aged 37 years (**h**). Corneal crystals (arrows) were present in all probands, although in proband 2 they were a minor feature (**b**), corresponding to an early stage of the disease, and in probands 4 and 5 (**g**, **h**) they were present in only a very small area (arrows). All images show findings in the right eye

Clinical, demographic and genotype data for all six probands are summarized in Table 1. There was no family history of SCD in two pedigrees; however, in family 3 the disease status of the proband’s mother was unavailable. The corneal phenotypes were diverse and included anterior and mid-stromal crystalline deposits, diffuse stromal haze and arcus lipoides (Fig. 1). There was an incremental accumulation of corneal deposits with age, and the corneal changes were symmetric in all individuals. There were corneal crystals in all probands, although these deposits were minimal in some individuals (Fig. 1 g, h). Corneal crystals were present at slit lamp examination of proband 2 at age 6 years, but neither parent had signs of corneal disease. The patient was re-examined at the age 8 years, when an increase in the corneal crystals was noted (Fig. 1b), but the visual acuity had remained unchanged (Table 1).

**Fig. 2:**
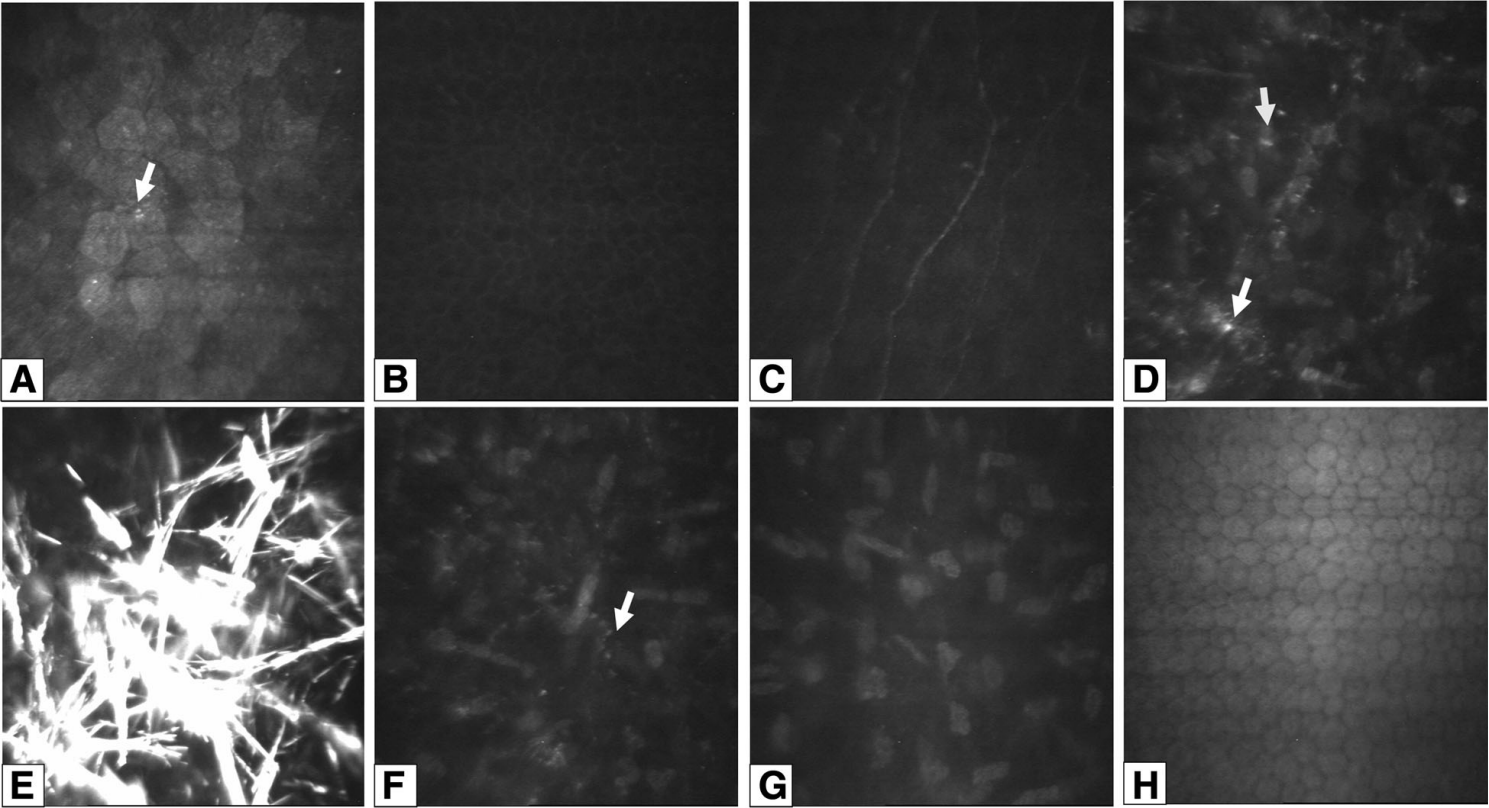
Corneal confocal microscopy imaging in an 8-year old child with Schnyder corneal dystrophy. Superficial epithelial cells with small round hyperreflective deposits (arrows) in the left eye (**a**). Normal appearance of the basal epithelial cell layer (**b**), and subepithelial nerve plexus in the right eye (**c**). Hyper-reflective deposits within and around keratocytes (arrows) (**d**) and needle-shaped crystals in anterior stroma of the left eye (**e**). Hyper-reflective deposits in mid-stroma in the left eye (**f**), but with unaffected posterior stroma (**g**) and endothelium in the left eye (**h**)

Corneal imaging highlighted the presence of crystals. With SD-OCT there were highly reflective deposits in the anterior stroma of probands 1 and 2 (Fig. 1c, d), with confocal microscopy there were bright reflective crystalline deposits identified in the anterior stroma of proband 2 (Fig. 2e). On confocal microscopy small round deposits were also identified in the superficial epithelial cells (Fig. 2a), in and around anterior stromal keratocytes (Fig. 2d) and in mid-stroma (Fig. 2f). The basal epithelial cells, sub-epithelial nerves, posterior stroma and corneal endothelium all appeared normal (Fig. 2b, c, g, h).

**Fig. 3:**
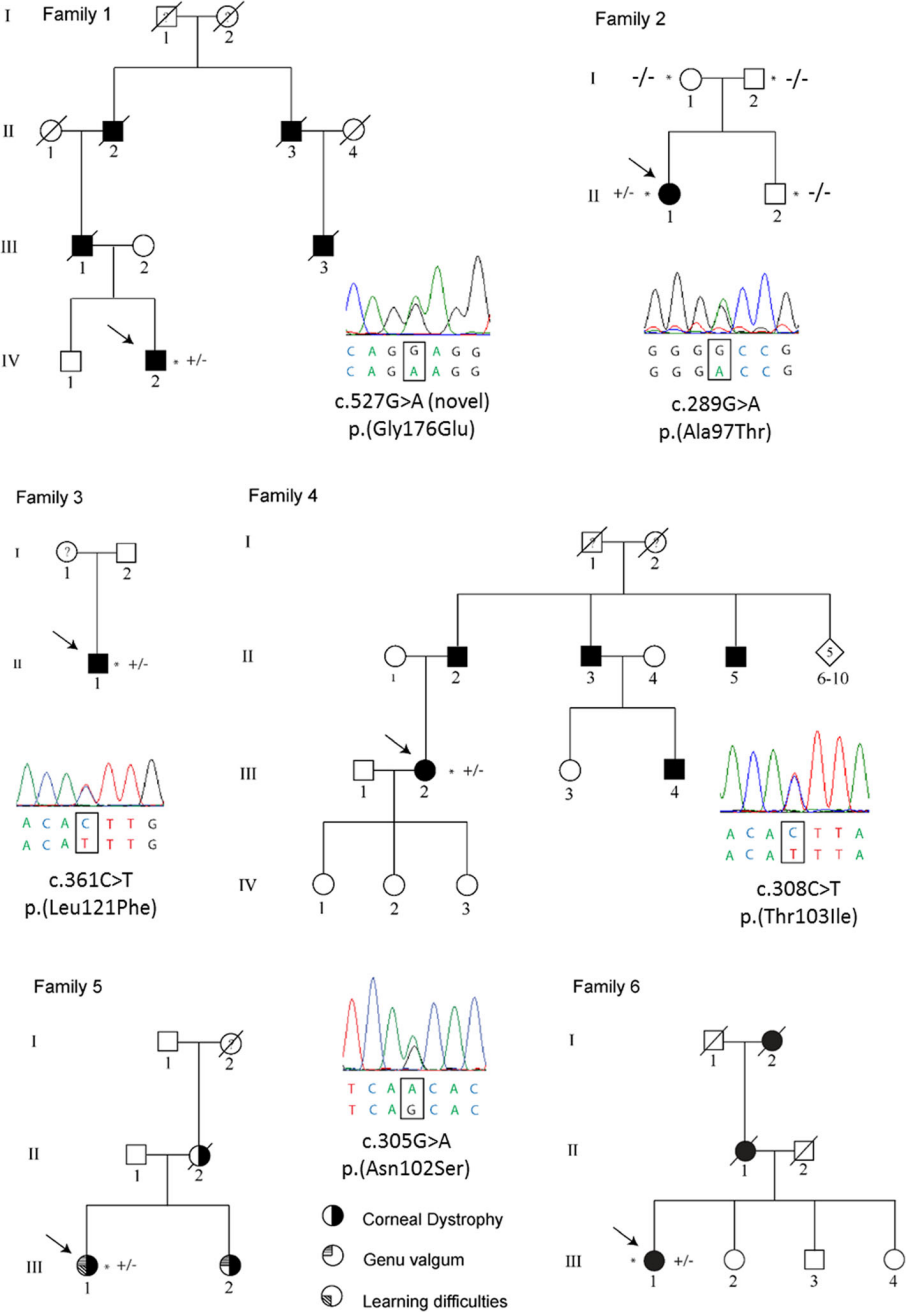
Pedigrees of the six families with Schnyder corneal dystrophy. Sequence electropherograms of the identified heterozygous mutations in *UBIAD1* are also shown. The mutation arose de novo in family 2. Probands are indicated by an arrow and examined individuals by an asterisk. Mutation status in tested subjects is shown +/− for those who are heterozygous for a mutation in *UBIAD1* and −/− for those who do not carry the pathogenic variant*.* Individuals known to be affected by Schnyder corneal dystrophy are shown in black, whereas a question mark indicates that the disease status of the individual was unknown

Coexisting systemic disease was present in some probands. Proband 5 and her affected sister both had bilateral knee deformities, although their affected mother was normal (Fig. 3). In proband 1 a fasting lipid profile showed high levels of total cholesterol (5.72 mmol/l; normal values in adults < 5.17 mmol/l) [16] and in probands 2 and 3 there was a borderline elevation of total cholesterol to 4.89 mmol/l and 5.00 mmol/l, respectively (normal values in children < 4.40 mmol/l) [17].

A novel c.527G>A; p.(Gly176Glu) variant, predicted to be pathogenic or probably pathogenic by all six bioinformatic tools (Table 3), was identified in proband 1 of Czech origin*.* The amino acid residue Gly-176 is highly conserved and located in the transmembrane domain of *UBIAD1*, therefore a mutation is likely to disrupt the transmembrane helices and active site [18]. Czech proband 2 harboured a known *UBIAD1* c.289G>A; p.(Ala97Thr) mutation. This variant was absent in both parents, suggesting a de novo origin that was confirmed by paternity testing. The white British probands had previously reported mutations c.361C>T; p.(Leu121Phe) [19] and c.308C>T; p.(Thr103Ile) [6]. Two reportedly unrelated British families of South Asian origin, both harboured a known c.305G>A; p.(Asn102Ser) mutation [19–21]. Pedigrees and segregation of the respective heterozygous *UBIAD1* mutations are shown in Fig. 3. None of the *UBIAD1* pathogenic changes found in the current study were observed in the gnomAD dataset or in the Czech control population. All of the in silico algorithms predicted that the detected mutations were pathogenic or likely pathogenic, except for SNP&GO prediction for previously reported variant c.361C>T; p.(Leu121Phe) [19] (Table 3).

**Table 3:** In silico analysis of *UBIAD1* missense variants identified in patients with Schnyder corneal dystrophy in the current study

	MutPred	Polyphen2	PROVEAN	SNP&GO	SIFT	MutationTaster
p.(Ala97Thr)	Disease	Probably damaging	Disease	Disease	Disease	Disease
p.(Leu121Phe)	Disease	Probably damaging	Disease	Benign	Disease	Disease
p.(Thr103Ile)	Possibly damaging	Probably damaging	Disease	Disease	Disease	Disease
p.(Asn102Ser)	Disease	Probably damaging	Disease	Disease	Disease	Disease
p.(Gly176Glu)	Possibly damaging	Probably damaging	Disease	Disease	Disease	Disease

Six different algorithms were used; tolerated and neutral scores are indicated in green as benign; yellow indicates a possibly damaging variant, and red was used for a probably damaging and disease-causing mutation

As for MutPred an overall probability score > 0.5 was considered as possibly damaging and a score > 0.75 was considered as disease-causing. NM_013319.2, NP_037451.1 and ENST00000376810.5 were used as reference sequences

## Discussion

In this study we report the phenotype and genotype of six families with SCD. Five different *UBIAD1* mutations were identified in a heterozygous state, of which one, c.527G>A; p.(Gly176Glu), was novel. The youngest proband was found to harbour a de novo c.289G>A; p.(Ala97Thr) mutation, previously identified in an Irish-French family [18]. To the best of our knowledge, this is only the second observation of a spontaneously occurring mutation in an SCD patient [6]. The family history provided by proband 3 also indicated possible de novo occurrence of the identified mutation, but unfortunately this could not be confirmed as parental DNA samples were unavailable.

The c.305G>A; p.(Asn102Ser) mutation, identified in two South Asian probands, is the most frequently occurring *UBIAD1* mutation. It has been reported in several populations including the Czech Republic, Poland, Taiwan and China, supporting the hypothesis that it is a mutation hotspot [19–22]. One white British proband had a c.361C>T; p.(Leu121Phe) mutation, previously observed in three SCD families from the UK, America and Saudi Arabia [19, 23]. The c.308C>T; p.(Thr103Ile) mutation, detected in one white British individual, has previously been described in a proband of Japanese-European descent [6].

The clinical course of SCD is associated with characteristic corneal opacities that increase with age. Initially, central corneal haze and/or crystals are present; this was observed in our youngest proband, who was 6 years old when first examined. Arcus lipoides typically develops in the third decade, followed by mid-peripheral corneal haze in the late fourth decade [24], as documented in the current case series (probands 1, 4–5). Corneal crystals are present in approximately 50% of patients with SCD [2]. Interestingly, crystals were found in all six of our probands, although in two probands the area of crystal deposit was very small. However, the number of individuals we examined is relatively low compared with prior studies [2].

Confocal microscopy has previously been performed in two children with SCD, both at a similar age as our proband 2 [25]. Our findings corroborate observations of accumulation of crystal/reflective material in anterior stroma, both intra- and extracellularly. Interestingly, unlike the previous study, subepithelial nerves appeared normal and we were able to detect tiny reflective deposits in the corneal epithelium. Electron microscopy of corneas with SCD has also documented lipid accumulation inside epithelial cells [26, 27].

The differential diagnosis of crystalline corneal deposition includes monoclonal gammopathy and cystinosis. These conditions should be considered in any individual with corneal crystals who does not have a family history of SCD. Laboratory investigation should be guided by the presence of associated symptoms and patient age.

Dyslipidemia and genu valgum have been reported to be associated with SCD [3, 28]. Three of the six probands in this study had fasting serum lipid testing. Total cholesterol was elevated in one proband and borderline levels were found in the other two probands. Self-reported knee deformities were only present in proband 5 and her sister, although their mother was not affected, which may indicate that other genetic or environmental factors influence the expression of this trait.

## Conclusions

SCD should be considered in the differential diagnosis of any unexplained corneal haze and/or crystal deposition, even in the absence of a family history of corneal disease.

## Abbreviations

BCVA: Best corrected visual acuity
gnomAD: Genome Aggregation Database
MIM: Mendelian Inheritance in Man
SCD: Schnyder corneal dystrophy
*UBIAD1*: UbiA prenyltransferase domain-containing protein
